# Branch-duct intraductal papillary mucinous neoplasm (IPMN): Are cyst volumetry and other novel imaging features able to improve malignancy prediction compared to well-established resection criteria?

**DOI:** 10.1007/s00330-022-08650-5

**Published:** 2022-03-11

**Authors:** Raffaella M. Pozzi Mucelli, Carlos Fernández Moro, Marco Del Chiaro, Roberto Valente, Lennart Blomqvist, Nikolaos Papanikolaou, Johannes-Matthias Löhr, Nikolaos Kartalis

**Affiliations:** 1 Department of Radiology Huddinge, Karolinska University Hospital, O-huset 42, 14186 Stockholm, Sweden; 2 Department of Clinical Science, Intervention, and Technology, Karolinska Institutet, O-huset 42, 14186 Stockholm, Sweden; 3 Department of Clinical Pathology and Cancer Diagnostics, Karolinska University Hospital, Huddinge, 141 86 Stockholm, Sweden; 4 Division of Pathology, Department of Laboratory Medicine, Karolinska Institutet, Alfred Nobels Allé 8, 141 52 Stockholm, Sweden; 5 Division of Surgical Oncology, Department of Surgery, University of Colorado, Anschutz Medical Campus, 12631 E 17th Ave #6117, Aurora, CO 80045 USA; 6 Department of Surgical and Perioperative Sciences, Umeå University, Daniel Naezéns väg, 907 37 Umeå, Sweden; 7 Department of Medical Radiation Physics and Nuclear Medicine, Karolinska University Hospital, Solnavägen 1, 17177 Stockholm, Sweden; 8 Department of Molecular Medicine and Surgery, Karolinska Institutet, L1:00, 17176 Stockholm, Sweden; 9 Computational Clinical Imaging Group, Centre for the Unknown, Champalimaud Foundation, Av. Brasília, Doca de Pedrouços, 1400-038 Lisbon, Portugal; 10 Department of Radiology, Royal Marsden Hospital and The Institute of Cancer Research, London, SM2 5NG UK; 11 Computational Biomedicine Laboratory (CBML), Foundation for Research and Technology Hellas (FORTH), 70013 Heraklion, Greece; 12 Department of Upper Abdominal Diseases, Karolinska Comprehensive Cancer Center, Karolinska University Hospital, Hälsovägen, 13, 141 57 Huddinge, Stockholm, Sweden

**Keywords:** Pancreatic intraductal neoplasm, Pancreatic carcinoma, Cysts, Logistic models, Magnetic resonance imaging

## Abstract

**Objectives:**

Current guidelines base the management of intraductal papillary mucinous neoplasms (IPMN) on several well-established resection criteria (RC), including cyst size. However, malignancy may occur in small cysts. Since branch-duct (BD) IPMN are not perfect spheres, volumetric and morphologic analysis might better correlate with mucin production and grade of dysplasia. Nonetheless, their role in malignancy (high-grade dysplasia/invasive cancer) prediction has been poorly investigated. Previous studies evaluating RC also included patients with solid-mass-forming pancreatic cancer (PC), which may affect the RC yield. This study aimed to assess the role of volume, morphology, and other well-established RC in malignancy prediction in patients with BD- and mixed-type IPMN after excluding solid masses.

**Methods:**

Retrospective ethical review-board-approved study of 106 patients (2008–2019) with histopathological diagnosis of BD- and mixed-type IPMN (without solid masses) and preoperative MRI available. Standard imaging and clinical features were collected, and the novel imaging features cyst-volume and elongation value [EV = 1 − (width/length)] calculated on T2-weighted images. Logistic regression analysis was performed. Statistical significance set at two-tails, *p* < 0.05.

**Results:**

Neither volume (odds ratio (OR) = 1.01, 95% CI: 0.99–1.02, *p* = 0.12) nor EV (OR = 0.38, 95% CI: 0.02–5.93, *p* = 0.49) was associated with malignancy. Contrast-enhancing mural nodules (MN), main pancreatic duct (MPD) ≥ 5 mm, and elevated carbohydrate antigen (CA) 19-9 serum levels (> 37 μmol/L) were associated with malignancy (MN OR: 4.32, 95% CI: 1.18–15.76, *p* = 0.02; MPD ≥ 5 mm OR: 4.2, 95% CI: 1.34–13.1, *p* = 0.01; CA19-9 OR: 6.72; 95% CI: 1.89 – 23.89, *p* = 0.003).

**Conclusions:**

Volume and elongation value cannot predict malignancy in BD- and/or mixed-type IPMN. Mural nodules, MPD ≥ 5 mm and elevated CA19-9 serum levels are associated with higher malignancy risk even after the exclusion of solid masses.

**Key Points:**

*• Novel and well-established resection criteria for IPMN have been evaluated after excluding solid masses.*

*• BD-IPMN volume and elongation value cannot predict malignancy.*

*• Main pancreatic duct ≥ 5 mm, mural nodules, and elevated carbohydrate antigen 19-9 levels are associated with malignancy.*

**Supplementary Information:**

The online version contains supplementary material available at 10.1007/s00330-022-08650-5.

## Introduction

Intraductal papillary mucinous neoplasms (IPMN) are increasingly recognized pancreatic cystic neoplasms (PCN), often incidentally detected on cross-sectional imaging (i.e., CT and/or MRI) performed for other reasons. They encompass a variety of entities with different biological behavior, ranging from low-grade dysplasia (LGD) up to high-grade dysplasia and invasive carcinoma (HGD/INV) [1]. IPMN may coexist with another pancreatic cancer (PC) precursor, such as pancreatic intraepithelial neoplasia (PanIn) [2]. Thus, IPMN necessitates surveillance and potentially surgical treatment to prevent pancreatic cancer (PC) [3, 4].

According to current guidelines, there are several features associated with risk for malignancy in patients with IPMN, with cyst size among them [3, 4]. However, cystic diameter still represents a controversial issue. There is indeed no agreement upon whether larger cysts may be associated with a higher risk of malignancy [5–8], and HGD/INV may be encountered even in smaller cysts [9]. Therefore, it is unclear whether the maximal cystic diameter can provide enough information for risk stratification.

A few studies investigated the role of cystic volume in the morphologic assessment of PCNs [10–12]. Since IPMNs are not perfect spheres, the largest diameter might not correctly represent the entire inner surface of the IPMN [11], whose epithelium is affected by varying grade of dysplasia up to invasive carcinoma. Hypothetically, IPMN volume would correlate better than size alone with the amount of secreted mucin in IPMN lesions, depending on their expression pattern of highly glycosylated proteins (MUC) [13] and their grade of dysplasia. Therefore, volumetry would then be helpful in stratifying IPMNs at risk of malignancy. Moreover, there are also other imaging features in IPMNs that would be of interest to explore regarding their impact on the prediction of malignancy. This includes morphologic features expressed by the relationship between width and length, defined in a previous paper as elongation value (EV) [11]. However, it is still unclear whether the shape of a cyst may play a role in predicting malignancy.

Furthermore, current guidelines recommend surgery based on other features, such as the dilatation of the main pancreatic duct (MPD), the elevation of serum level of the tumor marker carbohydrate antigen (CA)19-9, the presence of mural nodules (MN) and the progression in size of the cystic neoplasm during surveillance [3, 4]. Recently, a nomogram including several imaging features (i.e., MPD and cyst diameter, presence of MN, cyst location) has been proposed for better understanding the malignancy risk of an individual and further personalize the treatment management [14].

Interestingly, most studies that analyze the impact of clinical and imaging features have included patients with solid-mass-forming PC, which may affect the results. In these cases, the obstructing effect of a solid mass on the MPD is very likely to represent the main cause of its dilatation. Thus, including these patients in the analysis may overestimate the positive yield of the parameter MPD dilatation and, therefore, the decision upon surgery versus surveillance in patients with dilated MPD in the setting of absence of a solid-mass-forming PC at preoperative imaging. However, excluding solid masses should not influence the relationship of the BD-IPMN’s diameter and its grade of dysplasia/invasiveness.

The aim of this study was to assess the role of volume, morphology, and all other well-established RC in malignancy prediction in patients with BD- and mixed-type IPMN after the exclusion of solid-mass-forming PC.

## Materials and methods

Retrospective single-center study approved by the regional ethical review board (EPN 2015/1544–31/4). Patient informed consent was waived.

### Study population

All patients were recruited consecutively from a prospectively collected database of patients who underwent pancreatic surgery at Karolinska University Hospital during the period 2008–2019 and had a histologically verified IPMN. The indication, type, and extent of surgery were determined at a multidisciplinary team conference for all patients following guidelines present at the time of surgery (“Sendai criteria” [15] until November 2012; “European experts consensus statement on cystic tumors of the pancreas” [16] from December 2012 until February 2018; EEG 2018 [4] from March 2018).

**Fig. 1:**
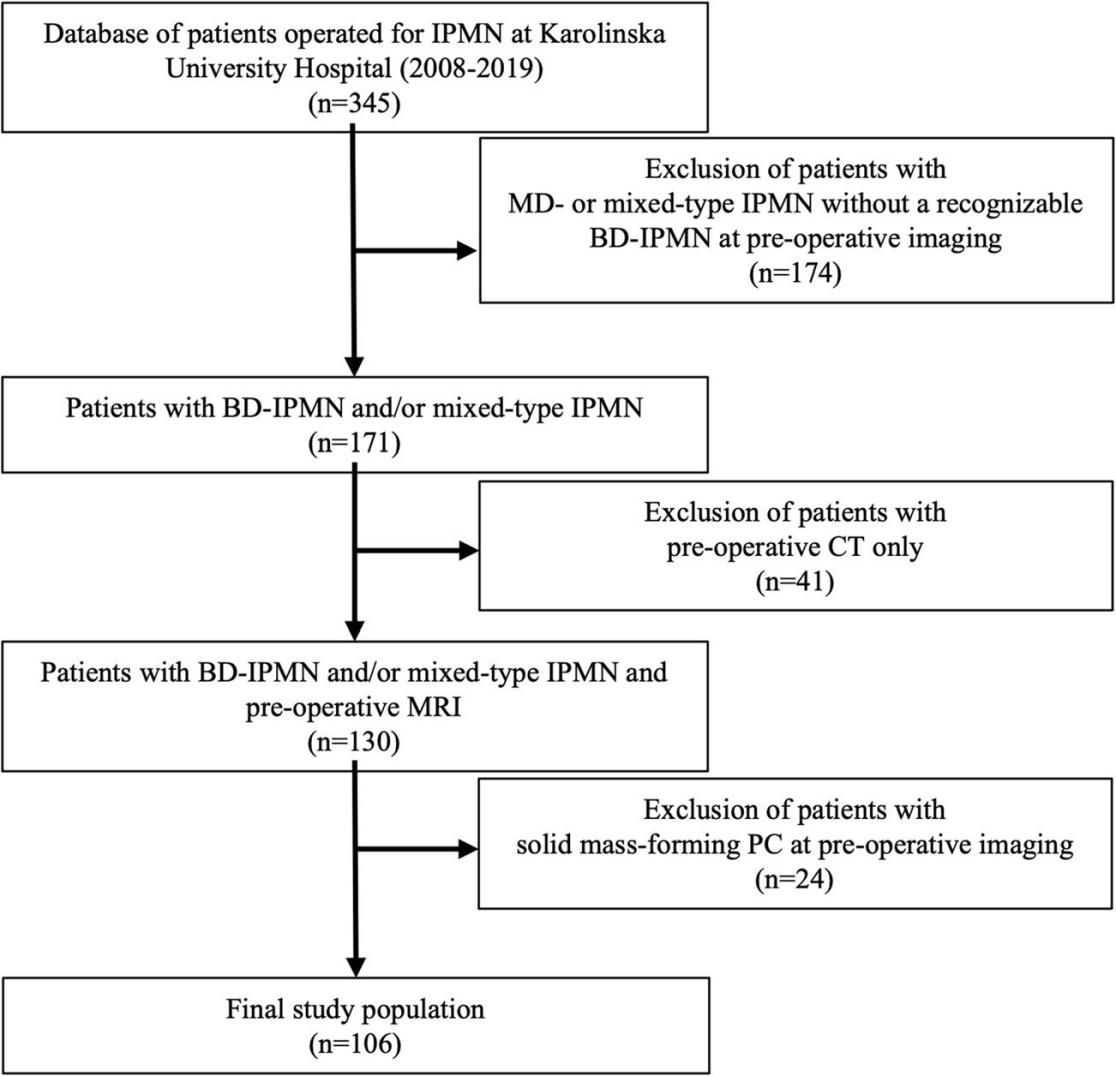
Flow chart showing the selection of study population (MD-: main-duct type; BD-IPMN: branch-duct IPMN; PC: pancreatic cancer)

The inclusion criteria were (a) preoperative pancreatic MRI with at least one axial and coronal T2-weighted sequence and (b) at least one histologically proven BD-IPMN detectable on the T2-weighted images (Fig. 1).

**Fig. 2:**
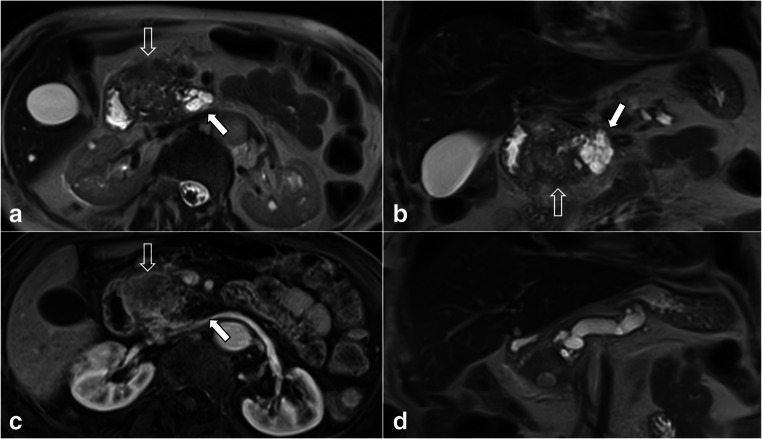
Pancreatic MRI of an 80-year-old patient with weight loss and abdominal pain. The axial (**a**) and coronal (**b**) T2-weighted images show a solid-mass-forming pancreatic cancer (PC) (open arrows) originating from an adjacent IPMN (white arrows) located in the head of the pancreas. The pancreatic cancer is homogeneously hypointense in the T1-w axial image in the pancreatic arterial phase (**c**). The mass-forming PC causes a stricture of the main pancreatic duct (MPD) with upstream dilatation on coronal T2-weighted image (**d**). The patient was excluded from our cohort, as the dilation of the MPD upstream secondary to a solid mass may lead to overestimation of the positive yield of the finding “dilated MPD”

The exclusion criteria were (a) main-duct diameter ≥ 5 mm without a BD-IPMN clearly identifiable at preoperative MRI and (b) solid-mass-forming PC with or without a MPD stricture (Figs. 1 and 2).

### Imaging analysis

The pancreatic MRI closest to the date of surgery was chosen for analysis. Since our institution is a tertiary high-volume center, some patients were referred for evaluation for surgery with outside MRI examinations using MR equipment from different vendors, sequences, and technical parameters. Therefore, an institutional standard protocol for preoperative MRI was not available for this study. The minimum criteria for including a non-institutional MRI in the study were (1) magnetic field strength ≥ 1.5 T; (2) availability of an axial and coronal T2-weighted sequence acquired with the single-shot technique (HASTE, Single-Shot Fast Spin Echo or Single-Shot Turbo Spin Echo) or multi-shot PROPELLER technique; and (3) slice thickness and interslice gap not larger than 6 mm and 20%, respectively.

All MR images were evaluated on a picture archiving and communication system (Sectra Workstation, IDS7 version 23.1, Sectra AB) by two radiologists in consensus reading (R.P.M. and N.K. with 15 and 12 years of post-residency experience in abdominal imaging, respectively). One cyst per patient was chosen for analysis (the largest or the one with the most suspicion for malignancy appearance based on current guidelines at the time of surgery). The collected imaging parameters are listed in Table 1.

**Table 1:** Collected imaging parameters

Imaging parameters	Description
Diameter 1 (Diam1)	Maximum cyst diameter on axial T2-weighted sequence (mm)
Diameter 2 (Diam2)	Maximum craniocaudal cyst diameter on coronal T2-weighted sequence (mm)
Cyst maximum diameter	Either Diam1 or Diam2, depending on which was largest (mm)
Elongation value (EV)	Defined as [1 − (width/length)] according to previous publication [11], where length was represented by the maximum diameter irrespective of the plane, and width as the maximum diameter perpendicular to length
Maximum MPD diameter	Expressed in mm
Mural nodules (MN)	Presence of contrast-enhancing mural nodules within the cyst
Cystic wall thickening	Present when cystic wall thickness ≥ 2 mm
Progress in size during follow-up	> 5 mm/year according to EEG 2018 [4]
Solitary/multifocal BD-IPMN	
Lesion localization	Head/uncinate process or body/tail
Cyst volume (Vsegm)	Calculated on axial T2-w images after file export to a free DICOM medical imaging viewer (Horos v2.1.1). A region of interest (ROI) was drawn along the edge of the BD-IPMN at multiple levels, using the tool “ROI volume” available in the semi-automatic three-dimensional segmentation software implemented in the viewer. The common bile duct and the MPD were excluded from the segmentation. Thereafter, the volume was automatically calculated by the software

*MPD* main pancreatic duct, *EEG* European evidence-based guidelines, *BD-IPMN* branch-duct IPMN

### Clinical features

From each patient’s electronic medical record, the following clinical parameters were collected: age at surgery, gender, presence of symptoms [such as jaundice, weight loss, abdominal pain, acute pancreatitis, recent (< 1 year) onset of diabetes mellitus] or incidentally discovered IPMN, elevated serum levels of CA 19-9 (> 37 μmol/L), and presence of familial/genetic predisposition to PDAC.

### Histopathological features

All the histopathological reports were examined, and the grade of dysplasia for the resected specimen recorded. In cases with an insufficient description of histopathological features [e.g., histotype, grade and location (i.e., cyst or MPD) of dysplasia], a side-by-side revision of the pathological specimen was performed by the pathologist (C.F.M.) in consensus with one radiologist (R.P.M.). No further systematic radiologic-pathologic correlation was performed.

### Statistical analysis

Normally and non-normally distributed variables were expressed by means and medians, respectively. Since the EEG 2018 use a certain cut-off for MPD diameter and serum levels of CA 19-9 in their recommendations [4], categorical variables for MPD and CA 19-9 were used for analysis. Wilcoxon rank sum test and chi-squared test were used to compare the outcome HGD/INV between two independent groups for numerical and categorical variables, respectively. Fisher’s exact test was applied when expected frequencies were less than 5. Univariable logistic regression analysis was performed to identify variables associated with the outcome HGD/INV. Odds ratios (OR) and 95% confidence intervals (CI) were calculated. Variables that were shown to be statistically significant at univariable logistic regression were tested with multivariable logistic regression (Enter Method), adjusted for age and gender. The predicted probabilities for the outcome HGD/INV were calculated for hypothetical male patients at age ≥ 70 years old with and without the variables that were shown to be statistically significant in multivariable logistic regression. The following diagnostic accuracy metrics of the solitary parameters were calculated: sensitivity, specificity, positive (PPV) and negative predictive values (NPV), and accuracy. A two-sided *p* value of < 0.05 was considered statistically significant. The statistical analysis was performed with Stata16 (StataCorp. 2019, Stata Statistical Software: Release 16, StataCorp LLC).

## Results

The final study population comprised 106 patients (Fig. 1). Of the 24 excluded patients (Fig. 1), 22 (92%) had a solid-mass-forming PC causing MPD stricture. Twenty-nine patients (27%) (operated on in the period 2008–2015) were part of the patient cohort in a previously published study [5] and 50 (47%) (operated on in the period 2008–2017) of the patient cohort in another study [6]. Patients’ characteristics are illustrated in Table 2. In our series, one-fourth of patients had HGD/INV (19/106 HGD and 8/106 invasive cancer). Among those eight patients with invasive cancer (one microinvasive), no visible mass-forming PC was detected pre-operatively. Fourteen patients had contrast-enhancing MN (mean size/range: 12/4–32 mm): 3 with high-grade dysplasia and 4 with invasive cancer (Table 2). The MN size was not statistically significantly different among patients with LGD and HGD/INV (*p* = 0.3). Seventy-eight patients (74%) were diagnosed with mixed-type IPMNs at surgical histopathology. The gastric type was the most prevalent histotype (70%) (Table 2).

**Table 2:** Characteristics of patients with branch duct (BD)–intraductal papillary mucinous neoplasms (IPMN) and mixed-type IPMN

Number of patients	106	Low-grade dysplasia	High-grade dysplasia/invasive cancer
Males	45/106 (42.4%)	31/45 (68.9%)	14/45 (31.1%)
Age (years)	Mean 68.2, median 70 (min 43, max 86)	Mean 67.9, median 70 (min 43, max 86)	Mean 68.8, median 70 (min 48, max 86)
Individuals at risk	3/106 (2.8%) (2 familiarity; 1 Peutz-Jeghers)	3/3 (100%)	0
Histology			
Low-grade dysplasia	79/106 (74.5%)		
High-grade dysplasia/invasive cancer	27/106 (25.5%)		8/106 inv.ca. (7.5%)
			8/27 inv.ca. (29.6%)
Mixed-type IPMN	78/106 (73.6%)	53/79 (67.1%)	25/27 (92.6%)
BD-IPMN at pre-op MRI	25/78 (32%)	21/53 (39.6%)	4/25 (16%)
Mixed-type IPMN at pre-op MRI	53/78 (68%)	32/53 (60.4%)	21/25 (84%)
Histological cell subtypes			
Gastric	75/106 (70.8%)	63/79 (79.8%)	12/27 (44.5%)
Pancreato-biliary (PB)	5/106 (4.7%)	2/79 (2.5%)	3/27 (11.1%)
PB + gastric	4/106 (3.8%)	3/79 (3.8%)	1/27 (3.7%)
Intestinal	7/106 (6.6%)	2/79 (2.5%)	5/27 (18.5%)
Intestinal + gastric	14/106 (13.2%)	8/79 (10.1%)	6/27 (22.2%)
PB + gastric + intestinal	1/106 (0.9%)	1/79 (1.3%)	0/27 (0%)
Symptoms^a^	32/106 (30.2%)	21/79 (26.6%)	11/27 (40.7%)
Jaundice	3/106 (2.8%)	1/79 (1.3%)	2/27 (7.4%)
Weight loss	3/106 (2.8%)	2/79 (2.5%)	1/27 (3.7%)
Abdominal pain	13/106 (12.3%)	9/79 (11.4%)	4/27 (14.8%)
Acute pancreatitis	15/106 (14.1%)	9/78 (11.4%)	6/27 (22.2%)
Diabetes (recent onset < 1 year)	0/53 (0%)		
Serum CA 19-9 (μmol/L)^b^	Median 11 (IQR 6–29) min 0.3, max 30359	Median 8.8 (IQR 4.8–21) min 0.3, max 60	Median 29 (IQR 10–74) min 1, max 30359
CA 19–9 > 37 μmol/L^b^	18/104 (17.3%)	9/77 (11.7%)	9/27 (33.3%)
IPMN localization			
Head/uncinate process	59/106 (55.6%)	42/79 (53.2%)	17/27 (62.9%)
Imaging features IPMN			
BD-IPMN at pre-op MRI	47/106 (44.3%)	41/79 (51.9%)	6/27 (22.2%)
Mixed-type IPMN at pre-op MRI	59/106 (55.7%)	38/79 (48.1%)	21/27 (77.8%)
Cyst max diameter (mm)	Median 33 IQR 24–42; min 9, max 100	Median 32 IQR 24–41; min 10, max 77	Median 36 IQR 24–47; min 9, max 100
Diameter ≥ 30 mm	65/106 (61.3%)	47/79 (59.5%)	18/27 (66.6%)
Diameter ≥ 40 mm	37/106 (34.9%)	25/79 (31.6%)	12/27 (44.4%)
Elongation value^c^	Mean 0.36 ± 0.16	Mean 0.37 ± 0.16	Mean 0.34 ± 0.16
Volume (cm^3^)	median 9.7 (IQR 4–19) min 0.3, max 424.2	median 9.4 (IQR 3–17) min 0.3, max 125.8)	median 11.4 (IQR 5–22) min 0.5, max 424.2
MPD max diameter (mm)	Mean 5.8 ± 3.3	Mean 5.3 ± 2.9	Mean 7.2 ± 4.1
	Median 5.1 (IQR 3.1–7.4) min 1.5, max 19	Median 4.9 (IQR 3–6.8) min 2, max 15	Median 6.6 (IQR 5.1–9.1) min 1.5, max 19
MPD ≥ 5 mm	59/106 (55.7%)	38/79 (48.1%)	21/27 (77.8%)
MPD 5–9.9 mm	48/106 (45.3%)	32/79 (40.5%)	16/27 (59.3%)
MPD ≥ 10 mm	11/106 (10.4%)	6/79 (7.6%)	5/27 (18.5%)
Contrast-enhancing mural nodules	14/106 (13.2%)	7/79 (8.9%)	7/27 (25.9%)
Size mural nodules (mm)	12.1 ± 7.6 (min–max 4–32)	9.2 ± 3.9 (min–max 5.3–17)	15 ± 9.6 (min–max 4–32)
Wall thickness ≥ 2 mm	6/106 (5.6%)	3/79 (3.8%)	3/27 (11.1%)
Solitary lesion	39/106 (36.8%)	30/79 (37.9%)	9/27 (33.3%)
Progress in size ( > 5 mm/year)	29/106 (27.4%)	24/79 (30.4%)	5/27 (18.5%)

*Pre-op* pre-operative, *MPD* main pancreatic duct

^a^Four patients had ≥ 2 symptoms

^b^Preoperative CA 19-9 was not available in two patients

^c^Elongation value calculated as [1 − (width/length)]

**Fig. 3:**
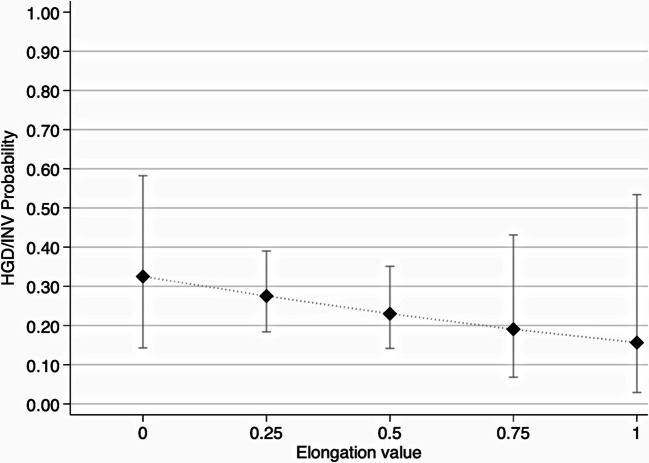
Two-way plot showing decreasing predicted probabilities and their 95% CI (*y*-axis) for the outcome high-grade dysplasia/invasive cancer (HGD/INV) over the elongation value (EV) (*x*-axis). The lower the EV (i.e., spheroid cyst), the higher the predicted probability of having HGD/INV and vice versa, although the variable did not result statistically significant at univariable logistic regression

Cyst volume was not statistically significantly different between patients with LGD and HGD/INV (*p* = 0.19). When analyzed in logistic regression (both alone and in combination with cystic EV), it was not associated with HGD/INV (Table 3). The mean EV was 0.36 (± SD 0.16), with a maximum value of 0.67 and an interquartile range of 0.25–0.5, showing that the majority of the segmented IPMN did not have a spheroid appearance (Table 2). At logistic regression, EV showed a tendency for inverse association with the outcome HDG/INV (OR = 0.38) although not statistically significant (Table 3). The predicted probabilities for the outcome HGD/INV slightly decreased by increasing the elongation value, although with broad confidence intervals (Fig. 3).

**Table 3:** Univariable logistic regression analysis for all clinical and imaging features

Patients’ features	Nr. of observations	Odds ratio	95% CI	*p* value*
Demographic and clinical features				
Age (years)	106	1.01	0.96–1.06	0.63
Age ≥ 70 (cohort’s median age)	106	1.05	0.44–2.51	0.91
Age < 70	106	0.95	0.39–2.28	0.91
Gender (male)	106	1.67	0.69–4.01	0.26
Localization (head/uncinate)	106	1.50	0.61–3.67	0.38
Mixed-type IPMN	106	6.13	1.34–27.89	0.02
Symptoms	106	1.90	0.76–4.74	0.17
Abdominal pain	106	1.35	0.38–4.81	0.64
Acute pancreatitis	106	2.22	0.71–6.97	0.17
Jaundice^a^	106	6.24	0.54–71.76	0.14
Weight loss	106	1.48	0.13–17.01	0.75
Serum CA 19-9 (μmol/L)	104	1.04	1.01–1.06	**0.002**
CA 19-9 > 37 μmol/L	104	3,77	1.30–10.9	**0.014**
Imaging-related features				
Volume (cm^3^)	106	1.01	0.99–1.02	0.12
Cyst max diameter (mm)	106	1.02	0.99–1.04	0.18
Diameter ≥ 30 mm	106	1.36	0.54–3.4	0.51
Diameter ≥ 40 mm	106	1.72	0.7–4.22	0.23
Elongation value	106	0.38	0.02–5.93	0.49
MPD max diameter (mm)	106	1.17	1.02–1.33	**0.02**
MPD ≥ 5 mm	106	3.97	1.45–10.89	**0.007**
MPD 5–9.9 mm	106	2.13	0.87–5.19	0.09
MPD ≥ 10 mm	106	2.76	0.77–9.93	0.12
Mural nodules	106	3.6	1.13–11.47	**0.03**
Wall thickness ≥ 2mm	106	3.16	0.59–16.73	0.17
Solitary lesion	106	0.81	0.32–2.05	0.66
Multifocal lesions	106	1.23	0.49–3.07	0.66
Progress in size (≥ 5 mm/year)^b^	67	1.01	0.36–2.8	0.98

*CI* confidence intervals, *CA* carbohydrate antigen, *MPD* main pancreatic duct

*A *p* value < 0.002 was considered statistically significant (marked in bold)

^a^No association was found between jaundice and elevated Ca19-9 (Fisher’s exact test, *p* = 0.56)

^b^Calculated on 67 observations (39 subjects had no previous examinations)

At univariable logistic regression analysis, maximum cyst diameter, wall thickness, solitary or multiple lesions and progress in size ( ≥ 5 mm/year) were not associated with HGD/INV (Table 3). The only variables associated with HGD/INV at univariable logistic regression were the presence of contrast-enhancing MN, diameter of the MPD ≥ 5 mm and serum levels of CA 19-9 > 37 μmol/L. This strong association was also confirmed in a multivariable logistic regression model adjusted for age and gender (Table 4). Histological cell subtypes did not correlate with cyst diameter, volume or EV (results not shown).

**Table 4:** Multivariable logistic regression analysis adjusted for age and gender

Patients’ features	Nr. of observations	OR	95% CI	*p* value*
Mural nodules	104	4.32	1.18 – 15.76	0.02
MPD ≥ 5 mm	104	4.2	1.34 – 13.1	0.01
CA19 - 9 > 37 μmol/L	104	6.72	1.89–23.89	0.003
Age at surgery (years)	104	1.01	0.95 – 1.07	0.61
Gender (male)	104	1.97	0.69 – 5.67	0.20

*A *p* value < .05 was considered statistically significant

**Fig. 4:**
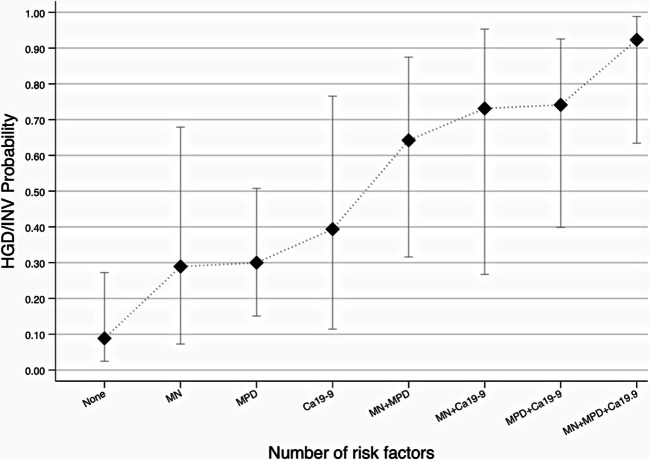
Two-way plot showing the predicted probabilities and their 95% CI (*y*-axis) for the outcome high-grade dysplasia/invasive cancer (HGD/INV) over the different combinations of risk factors (*x*-axis) for a hypothetical male patient with age ≥ 70 years old. Predicted probabilities were estimated by a multivariable logistic regression model, as described in the section “Materials and methods.” Abbreviations: MN: contrast-enhancing mural nodules, MPD: main pancreatic duct diameter equal to or larger than 5 mm; CA19-9: carbohydrate antigen 19-9 levels higher than 37 μ/μmol/L

**Fig. 5:**
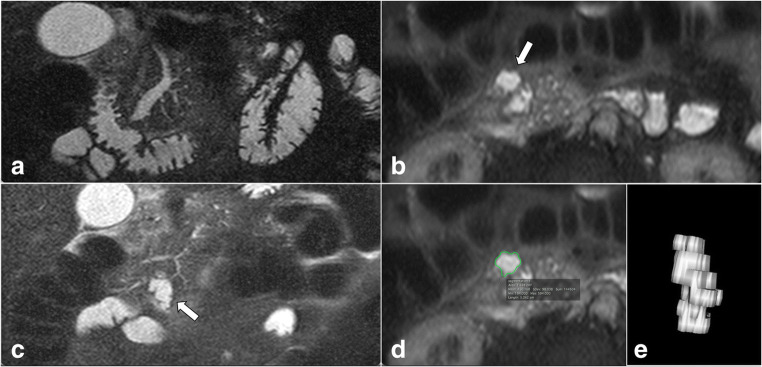
Pancreatic MR images of a 61-year-old man with recurrent episodes of acute pancreatitis. The main pancreatic duct (MPD) diameter is 9 mm in the head of the pancreas on coronal T2-weighted image (**a**), and a branch-duct intraductal papillary mucinous neoplasm (BD-IPMN; white arrows) is identified anteriorly in the uncinate process on axial (**b**) and coronal (**c**) T2-weighted images. The BD-IPMN was segmented using Horos v2.1.1 (**d**), and a volume of approximately 5 cm^3^ was obtained (**e**). Due to the presence of suspected IPMN–related acute pancreatitis, MPD diameter larger than 5 mm and elevated carbohydrate antigen 19-9 levels (80 μmol/L), the patient underwent pancreaticoduodenectomy. The final histopathological diagnosis was mixed-type IPMN with high-grade dysplasia

The predicted probabilities calculated for a hypothetical male patient with age ≥ 70 years old progressively increased by adding the risk factors contrast-enhancing MN, diameter of the MPD ≥ 5 mm and serum levels of CA 19-9 > 37 (Fig. 4). Namely, the predicted probability for the outcome HGD/INV with none of the aforementioned risk factors was 0.08 and increased to 0.92 when all of the risk factors were present. Table 5 shows observed probabilities for the outcome HGD/INV in the cohort’s patients depending on the number of positive risk factors. Figure 5 presents a case of a patient operated on for suspected mixed-type IPMN with dilated MPD and elevated CA 19-9, with final histology of HGD.

**Table 5:** Observed probabilities for the outcome high-grade dysplasia/invasive cancer (HGD/INV) versus low-grade dysplasia (LGD) in the cohort’s patients depending on the presence of risk factors contrast-enhancing mural nodules (MN), main pancreatic duct diameter equal to or larger than 5 mm (MPD), and carbohydrate antigen 19-9 levels higher than 37 μmol/L (CA 19-9)

LGD versus HGD/INV	Sum of observed risk factors (MN, MPD, CA19-9)				
	0	1	2	3	Total
LGD	91.4% (32/35)	77.4% (41/53)	35.3% (6/17)	0	74.5% (79/106)
HGD/INV	8.6% (3/35)	22.6% (12/53)	64.7% (11/17)	100% (1)	25.5% (27/106)

Interestingly, an MPD ≥ 5 mm represented the only surgical indication in 15 patients, of whom three were diagnosed with HGD/INV (20%) (sensitivity 11.1% (95% CI: 2.3–29%; *n* = 3/27) and positive predictive value (PPV) of 20% (95% CI: 4.3–48%; *n* = 3/15)). The diagnostic metrics for all resection criteria taken alone are reported in Supplementary Material Table S1.

## Discussion

Our results indicate that cystic volume as well as other imaging features, such as the maximum cystic diameter, wall thickness and solitary/multifocal lesions, as well as progress in size (≥ 5 mm/year), failed in predicting HGD/INV in patients operated on for BD- and mixed-type IPMN.

To the best of our knowledge, only one previous paper attempted to evaluate the role of tumoral volume in the prediction of malignancy in patients with IPMN [10], showing that an intraductal volume of ≥ 10 cm^3^ had a sensitivity and specificity of 70% and 73% in diagnosing malignant IPMN. However, this paper included both CT and MRI scans without stringent definitions of inclusion criteria. Moreover, the IPMN’s segmentation process was performed manually, based on a “by-pen” tracing method on paper that eventually was scanned and digitalized, rendering this method not feasible for routine clinical practice. Our semi-automatic segmentation method is more practical, although not correlated to the grade of dysplasia, and volumetry may potentially be performed with any segmentation tool available in any PACS system in a non-time-consuming fashion as proposed by Pandey P. et al [12]. Furthermore, semi-automatic volumetric segmentation has the important advantage of being independent of the axis manually chosen by the radiologist and has very high intra- and interobserver reproducibility even in smaller lesions [12, 17]. Thus, it may potentially overcome the issue of non-standardized manual measurements of cystic diameters, which are affected by intra- and inter-observer variability [17].

We also hypothesized that the morphology of a BD-IPMN expressed by the EV might be associated with malignancy. For instance, a BD-IPMN with spheric appearance (i.e., EV close to zero) might be characterized by a greater mucin secretion, depending on its MUC expression patterns and grade of dysplasia. Although the increase in EV appears slightly inversely associated with the risk of HGD/INV (OR 0.38) per one-unit increase, this association was not statistically significant. Thus, there is no sufficient evidence to support the hypothesis that the morphology of BD-IPMN may be associated with HGD/INV.

The presence of contrast-enhancing MN showed an association with HGD/INV. Despite the low prevalence of this parameter in our cohort (14/106, 13%), seven patients with contrast-enhancing MN were diagnosed with HDG/INV. This is in line with other papers and, more recently, with the systematic review and meta-analysis published by Marchegiani G. et al [18].

Interestingly, MPD dilatation was another imaging-related factor associated with a higher risk of malignancy in patients with IPMN without a solid-mass-forming PC. We decided to evaluate the effect of the MPD dilatation in the absence of detectable solid masses at preoperative MRI, which very likely caused the obstruction of the MPD and its upstream dilatation. In this way, it was possible to analyze the real impact of MPD dilatation. Thus, as demonstrated by others [5, 6, 19], MPD dilatation appears to play an important role in terms of increased risk of HGD/INV in a surgical series, especially in association with other imaging and/or clinical risk factors, such as contrast-enhancing MN and elevated serum levels of CA 19-9. However, when the indication for surgery was MPD dilatation alone (15 patients), the sensitivity and positive predictive values were low (11% and 20%, respectively). This might be explained by the fact that the MPD dilatation in mixed-type IPMN is not exclusively related to diffuse malignant epithelial changes but may result from passive distension due to mucin secretion from solitary or multiple BD-IPMN. Thus, in patients with mixed-type IPMN, MPD dilatation as a sole resection criterion has to be carefully considered before the decision to proceed to surgery is taken.

Nonetheless, due to the small sample size, it was not possible to assess the impact of subclasses of MPD dilatation (i.e., MPD 5–9.9 mm and ≥ 10 mm). It is also necessary to underline that this study cohort excluded all patients with main-duct IPMN since the main aim was the volumetric and morphological analysis of BD-IPMNs.

The only clinical feature correlated to a higher risk of HGD/INV was the elevated serum level of CA 19-9. The presence of symptoms was not associated with HGD/INV in our cohort. Mucin-producing tumors such as IPMN may cause abdominal pain and/or acute pancreatitis, which were the most often encountered symptoms in our cohort, and for these reasons, they are included among worrisome features and relative surgical indications in the current guidelines [3, 4]. Possible reasons for symptoms not being associated with malignant IPMN are the small sample size and the fact that these symptoms are often secondary to solid-mass-forming PC (an exclusion criterion in this study). The absence of pre-operatively detectable solid masses may even explain the low prevalence of jaundice in our patient cohort (3/106, 2.8%).

The main strength of our study is represented by the selected population since, albeit small, it did not include patients with a suspected solid tumor at preoperative MRI. On the one hand, the presence of a solid mass associated with an IPMN preoperatively is a major indication for surgery and potentially a very late stage of IPMN malignant transformation (thus, beyond the aim of preventive surgery). On the other hand, pooling together subjects with IPMN and solid masses causing a MPD stricture with upstream dilatation (Fig. 2) may lead to overestimation of the yield of the risk factor “MPD dilatation”. In daily praxis, it is very common to encounter patients with imaging-related risk factors (e.g., dilatation of the MPD, enlarged BD-IPMN, MN, etc.) and no detectable solid mass. In these cases, the appropriate assessment of the risk-benefit of surgery is mainly based on imaging and clinical features according to previously published data, that if influenced by major suspected features (i.e., pancreatic mass causing MPD obstruction), may possibly lead to wrong decisions.

Our study has several limitations. The main ones are its retrospective nature and the fact that it only included operated patients with a diagnosis of IPMN. This is an unavoidable and well-known limitation for all studies investigating similar topics and affects the possibility of evaluating the real performance of our findings on a “population-level” basis. Furthermore, the study cohort is small. However, we still had statistically significant results. For a more precise estimation of the role of new imaging-related risk factors in patients with IPMN, larger samples are probably needed. Additionally, the indications for surgery have varied during the span of the study, which may have slightly affected our results. Furthermore, we did not correlate MN to the exact location of HGD/INV-focus. Another limitation is the use of T2-w axial images for the segmentation and volumetry of BD-IPMN that may lead to less precise measurements. However, 3D-MRCP sequences were not available in all patients, and, when available, the presence of artifacts oftentimes did not permit any measurements. Nonetheless, 3D-MRCP sequences are not considered necessary for the assessment of resection criteria [4]. In case of artifacts, the more robust 2D-MRCP may be used, but it is not suitable for computing volumetry. Additionally, the MR images were evaluated by two radiologists in consensus. For this reason, it was not possible to assess the inter-observer agreement regarding the measured parameters.

Finally, we included MRI examinations from different vendors and protocols. However, this increases the generalisability of our results.

In conclusion, our study shows that neither volumetry nor other novel imaging features of BD-IPMN can predict malignancy. The dilatation of MPD, especially in conjunction with contrast-enhancing MN and abnormally elevated serum levels of CA 19-9, is associated with a higher risk of malignancy even when solid-mass-forming PC are excluded.

## Supplementary Information

ESM 1 (DOCX 20.6 KB)

## Abbreviations

BD: Branch duct
EEG: European evidence-based guidelines
EV: Elongation value
HGD/INV: High-grade dysplasia and invasive carcinoma
IPMN: Intraductal papillary mucinous neoplasm
LGD: Low-grade dysplasia
MN: Mural nodule
MPD: Main pancreatic duct
PC: Pancreatic cancer
PCN: Pancreatic cystic neoplasm
Vsegm: Cyst volume calculated by segmentation

## Declarations

### Guarantor

The scientific guarantor of this publication is associate professor Nikolaos Kartalis.

### Statistics and Biometry

Nicola Orsini, associate professor of Medical Statistics, head of the Biostatistics Team, Department of Global Public Health, kindly provided statistical advice for this manuscript.

### Study subjects or cohorts overlap

The final population of our study overlaps with two previously published papers.

Twenty-nine patients out of 106 (27%) (operated on in the period 2008–2015) were part of the patient cohort in a previously published study by our group [reference nr. 5: Ateeb Z, Valente R, Pozzi-Mucelli RM, et al (2019) Main pancreatic duct dilation greater than 6 mm is associated with an increased risk of high-grade dysplasia and cancer in IPMN patients. Langenbecks Arch Surg].

Fifty out of 106 patients (47%) (operated on in the period 2008–2017) were part of the patient cohort in the study by Del Chiaro M. et al [reference nr. 6: del Chiaro M, Beckman R, Ateeb Z, et al (2019) Main duct dilatation is the best predictor of high-grade dysplasia or invasion in intraductal papillary mucinous neoplasms of the pancreas. Ann Surg].

Our study differs from the previous paper from Ateeb Z et al and del Chiaro et al as we only included patients with a preoperative pancreatic MRI, who had at least one histologically proven BD-IPMN detectable on the T2-weighted images. Moreover, the main aim of our current study was to assess the role of cystic volume, morphological features such as elongation value and other well-established resection criteria after (1) excluding subjects with solid-mass-forming IPMN–associated or concomitant pancreatic cancer, and (2) patients with main-duct diameter ≥ 5 mm without a BD-IPMN clearly identifiable at preoperative MRI.

### Methodology

- retrospective
- cross-sectional diagnostic study
- performed at one institution
