# Publisher Correction: Branch-duct intraductal papillary mucinous neoplasm (IPMN): Are cyst volumetry and other novel imaging features able to improve malignancy prediction compared to well-established resection criteria?

**DOI:** 10.1007/s00330-022-09309-x

**Published:** 2022-12-13

**Authors:** Raffaella M. Pozzi Mucelli, Carlos Fernández Moro, Marco Del Chiaro, Roberto Valente, Lennart Blomqvist, Nikolaos Papanikolaou, Johannes-Matthias Löhr, Nikolaos Kartalis

**Affiliations:** 1 Department of Radiology Huddinge, Karolinska University Hospital, O-huset 42, 14186 Stockholm, Sweden; 2 Department of Clinical Science, Intervention, and Technology, Karolinska Institutet, O-huset 42, 14186 Stockholm, Sweden; 3 Department of Clinical Pathology and Cancer Diagnostics, Karolinska University Hospital, Huddinge, 141 86 Stockholm, Sweden; 4 Division of Pathology, Department of Laboratory Medicine, Karolinska Institutet, Alfred Nobels Allé 8, 141 52 Stockholm, Sweden; 5 Division of Surgical Oncology, Department of Surgery, University of Colorado, Anschutz Medical Campus, 12631 E 17th Ave #6117, Aurora, CO 80045 USA; 6 Department of Surgical and Perioperative Sciences, Umeå University, Daniel Naezéns väg, 907 37 Umeå, Sweden; 7 Department of Medical Radiation Physics and Nuclear Medicine, Karolinska University Hospital, Solnavägen 1, 17177 Stockholm, Sweden; 8 Department of Molecular Medicine and Surgery, Karolinska Institutet, L1:00, 17176 Stockholm, Sweden; 9 Computational Clinical Imaging Group, Centre for the Unknown, Champalimaud Foundation, Av. Brasília, Doca de Pedrouços, 1400-038 Lisbon, Portugal; 10 Department of Radiology, Royal Marsden Hospital and The Institute of Cancer Research, London, SM2 5NG UK; 11 Computational Biomedicine Laboratory (CBML), Foundation for Research and Technology Hellas (FORTH), 70013 Heraklion, Greece; 12 Department of Upper Abdominal Diseases, Karolinska Comprehensive Cancer Center, Karolinska University Hospital, Hälsovägen, 13, 141 57 Stockholm, Huddinge Sweden

**Publisher Correction: European Radiology (2022) 32:5144-5155**

10.1007/s00330-022-08650-5

The original version of this article, published on 11 March 2022, unfortunately contained a mistake. Due to a typesetting error, the wrong electronic supplementary file was uploaded and has now been replaced. The original article has been corrected.

## Supplementary Information

ESM 1 (DOCX 20 kb)

