# Supplementary material for: Branch-duct intraductal papillary mucinous neoplasm (IPMN): Are cyst volumetry and other novel imaging features able to improve malignancy prediction compared to well-established resection criteria?
Source: Eur Radiol. 2022 Mar 11;32(8):5144–55. doi: 10.1007/s00330-022-08650-5 (PMC9279268; doi:10.1007/s00330-022-08650-5)
Supplement: Supplementary file 1 — (DOCX 20.6 KB) [file 330_2022_8650_MOESM1_ESM.docx]

***Supplementary material***

**Table S1.** Performance of imaging and clinical features included in the European Evidence-based Guidelines^a^ as “*solitary*” risk factors

| **Surgical indications** | **Sensitivity % [95%CI], (n)** | **Specificity % [95%CI], (n)** | **PPV % [95%CI], (n)** | **NPV % [95%CI], (n)** | **Accuracy % [95%CI], (n)** |
| --- | --- | --- | --- | --- | --- |
| **Clinical features** |  |  |  |  |  |
| Symptoms | 3.7 [0.09-18.9] (1/27) | 94.9 [87.5.98.6] (75/79) | 20 [0.5-71.6] (1/5) | 74.2 [64.5-82.4] (75/101) | 71.7 [62.1-80] (76/106) |
| - Acute pancreatitis | 0 | 97.5 [91.1-99.6] (70/79) | 0 | 74 [64.5-82.1] (77/104) | 72.6 [63.1-80.8] (77/106) |
| - Jaundice | 3.7 [0.09-18.9] (1/27) | 100 [95.4-100] (79/79) | 100 [2.5-100] (1/1) | 75.2 [65.8-83.1] (79/105) | 75.5 [66.1-83.1] (80/106) |
| - Diabetes (recent onset) | 0 | 0 | 0 | 0 | 0 |
| CA 19-9 >37 𝜇mol/L | 0 | 0 | 0 | 0 | 0 |
| **Imaging-related features** |  |  |  |  |  |
| Diameter ≥ 40 mm | 3.7 [0.09-18.9] (1/27) | 92.4 [84.2-97.2] (73/79) | 14.3 [0.36-57.8] (1/7) | 73.7 [63.9-82.1] (73/99) | 69.8 [60.1-78.3] (74/106) |
| MPD 5-9.9 mm | 11.1 [2.3-29] (3/27) | 84.8 [74.9-91.8] (67/79) | 20 [4.3-48 (3/15) | 73.6 [63.3-82.3] (67/91) | 66 [56.1-74.9] (70/106) |
| MPD ≥ 10 mm | 0 | 0 | 0 | 0 | 0 |
| Contrast-enhancing MN | 0 | 96.2 [89.3-99.2] (76/79) | 0 (0/3) | 73.8 [64.2-81.9] (76/103) | 71.7 [62.1-80] (76/106) |
| Progress in size (≥ 5mm/year) | 0 | 87.3 [77.9-93.7] (69/79) | 0 (0/10) | 71.8 [61.7-80.6] (69/96) | 65.1 [55.2-74.1] (69/106) |

^a^The European Study Group on Cystic Tumours of the Pancreas (2018) European evidence-based guidelines on pancreatic cystic neoplasms. Gut 67:789–804. https://doi.org/10.1136/gutjnl-2018-316027

Abbreviations: MPD: main pancreatic duct. MN: mural nodules.
